# Developing a Patient-Centered mHealth App: A Tool for Adolescents With Type 1 Diabetes and Their Parents

**DOI:** 10.2196/mhealth.6654

**Published:** 2017-04-19

**Authors:** Bree E Holtz, Katharine M Murray, Denise D Hershey, Julie K Dunneback, Shelia R Cotten, Amanda J Holmstrom, Arpita Vyas, Molly K Kaiser, Michael A Wood

**Affiliations:** ^1^ Department of Advertising and Public Relations Michigan State University East Lansing, MI United States; ^2^ College of Nursing Michigan State University East Lansing, MI United States; ^3^ Sparrow Health System Department of Pediatric Endocrinology Lansing, MI United States; ^4^ Department of Media and Information Michigan State University East Lansing, MI United States; ^5^ Department of Communication Michigan State University East Lansing, MI United States; ^6^ Department of Pediatrics Texas Tech University Lubbock, TX United States; ^7^ Department of Pediatrics University of Michigan Ann Arbor, MI United States

**Keywords:** mHealth, qualitative research, type 1 diabetes, family

## Abstract

**Background:**

Type 1 diabetes (T1D) afflicts approximately 154,000 people under 20 years of age. Three-quarters of adolescents are not achieving glycosylated hemoglobin (HbA1c) targets, which leads to negative health outcomes. Mobile health (mHealth), the use of technology in health, has been used successfully to improve health in many chronic conditions, including diabetes.

**Objective:**

The purpose of this study was to use patient-centered research methods to inform and improve the design and functionality of our T1D app, *MyT1DHero,* and to provide insight for others who are designing a health app for adolescents and parents.

**Methods:**

This study included data from focus groups with participants recruited from the Juvenile Diabetes Research Foundation (JDRF) southeast Michigan’s family network. All data collected during the sessions were audio-recorded, transcribed, and coded.

**Results:**

Four key themes were identified: (1) diabetes is unpredictable, (2) negative and frustrated communication, (3) motivations to use an app, and (4) feedback specific to our app.

**Conclusions:**

A patient-centered approach was used to assist in the development of an app for adolescents with T1D. Participants were satisfied with overall app design; customization, interactivity, and tangible rewards were identified as being necessary for continued use. Participants believed the app would help improve the communication between parents and adolescents. Many apps developed in the health context have not used a patient-centered design method or have seen vast improvements in health. This paper offers suggestions to others seeking to develop apps for adolescents and their parents.

## Introduction

### Type 1 Diabetes

Type 1 diabetes (T1D) afflicts more than half a million children aged 0-14 years worldwide [1]. Many individuals are diagnosed with T1D at a young age, which requires parents to take responsibility for managing their child’s chronic health condition [2]. Proper management includes monitoring multiple daily blood glucose measurements, physical activity, carbohydrate intake, and adjusting insulin doses with the use of multiple daily insulin injections or an insulin pump for basal and bolus insulin delivery [3,4]. Glycemic control is monitored quarterly at medical visits with the glycosylated hemoglobin (HbA1c) test. As children become teenagers, they steadily transition toward self-management, gaining more responsibility for their T1D care, and their parents gradually relinquishing control [5]. This transition period is often difficult because of the complexity of managing T1D in the context of suboptimal communication between the child and parent, often resulting in deleterious health outcomes [6]. Inadequate management during this transition increases the risk of severe hypoglycemia, which can lead to seizures or coma, and ketoacidosis, which can cause neurologic sequelae and even death [7,8]. The long-term consequences of poor diabetes management include cardiovascular disease, stroke, kidney disease, blindness, amputation, and premature death [7-9]. Successful transition is critical to the health and well-being of the teen. Identifying interventions to aid in the transition of care can ultimately increase positive health outcomes and the quality of life for adolescents with T1D [10].

As a result of the complexity of managing T1D, there is often a lack of adherence to proper T1D self-care, resulting in an unsuccessful transition [11]. Approximately 75% of teens are not achieving American Diabetes Association (ADA) HbA1c targets during this transition period [12]. Therefore, identifying and finding solutions to help adolescents attain the knowledge and skills needed to succeed in diabetes management is imperative to increasing positive health outcomes [13]. To ease the transition, interventions that aim to improve productive communication between parents and teens, help build trust and autonomy, and develop problem-solving skills which have demonstrated improved diabetes outcomes [14]. Unfortunately, parent-teen communication is difficult during this transition time, particularly when interactions regarding their diabetes management are often negative [15]. For instance, teens feel their parents are nagging them about their diabetes care [16]. Infusing positive communication into the parent-child relationship is fundamental to the success of this difficult transition [17]. Building trust and autonomy is also important to allow the parent to take a step back from care and give responsibility of disease management to the teen. Finally, problem-solving skills for both the parent and the teen can further improve communication and trust, ultimately improving diabetes outcomes.

### mHealth

Mobile health (mHealth), the use of mobile technology in health, has been used in many health contexts, including asthma, pain management, depression, pregnancy, and others for more than a decade and has demonstrated positive outcomes [18-26]. Despite the proliferation of these health apps (over 165,000 available in Google Marketplace and Apple Store), very few have been developed using a patient-centered design [27]. The use of mHealth in diabetes care has been reported as useful in improving trends, but interventions have not been tested over a period of time sufficient to determine long-term engagement [28,29]. A recent study has called for patients to be more engaged in the design and development process [30]. Other studies have explored the wants and needs of an app for people with diabetes. Researchers have found that ease of use, timely and accessible information, and good visual appearance of the technology are all important [31,32]. Building an mHealth intervention using a patient-centered process to increase communication, trust, and autonomy should facilitate the goals of a successful transition to independence and achieving positive health outcomes and quality of life.

Examining the literature regarding participatory design within T1D and apps, we were unable to find any research that reported including both the parents and the child in the design. Previous research describes working with clinicians and developers [33,34], adults with T1D [35,36], or only the adolescents with T1D (no discussion with the parents) [19]. However, parent-child communication is critical during the transition to self-care and should be of central concern when designing an app for this context. Thus, our study focuses on communication that occurs between the parent and their child surrounding the management of diabetes, as it relates to the development of our app. Our expectation is that an app focused on easing the difficulties of diabetes-specific communication will result in positive health outcomes and better quality of life for both adolescents and their parents [37]. Therefore, we sought to better understand the perspective of both groups and gather feedback regarding our prototype.

The objective of this study was to use patient-centered research methods to inform and improve the design and functionality of our type 1 diabetes app, *MyT1DHero*. Additionally, we hope to provide insight for others who are designing a health app for adolescents and parents. To inform development of the app, we conducted qualitative focus groups to further explore how teens with T1D and their parents communicate about diabetes, what motivates them to use different apps and games, and evaluate their perceptions of our proposed app.

### App Description

**Figure 1:**
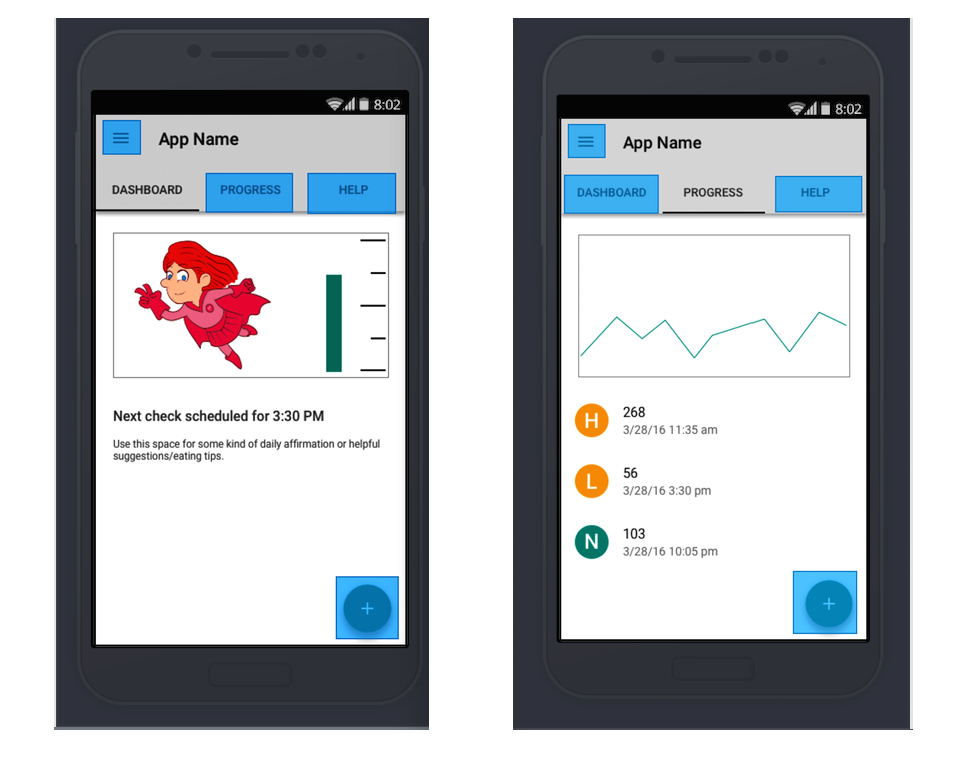
App screenshots.

*MyT1DHero* is a self-management mobile app that aims to encourage adolescents aged 10-15 years to self-manage their diabetes by recording their blood glucose (BG) values while allowing the parents seamless access to these same values. See Figure 1 for example screenshots of the app. The parent users have a separate login that allows them to access the app and review their child’s BG value history from their own phone. The parent and adolescent may also exchange messages via the app that are geared to facilitate positive communication. In addition, the app will have customizable test reminders, BG ranges, and an educational component that provides diabetes information and social support.

The adolescents will also earn points based on app usage. The accumulated points will allow for the adolescent to purchase “accessories” to customize their avatar, or a virtual character on the app.

## Methods

### Recruitment and Participants

Participants were recruited via the Juvenile Diabetes Research Foundation (JDRF) southeast Michigan’s family network, including Facebook, Twitter, and their email listserv. Inclusion criteria included: the adolescents must: (1) have a T1D diagnosis according to the ADA practice guidelines, (2) be 10-15 years old, (3) have had a diagnosis of T1D for at least six months, and (4) be fluent in English. The parent or guardian must: (1) have an adolescent with T1D who is 10-15 years old and who has had a diagnosis for at least 6 months and (2) be fluent in English. The study was approved by the Michigan State University Institutional Review Board. There were 5 adolescent participants in the focus group aged 10-13 years. The parent focus group had 7 participants, each with a child having a diagnosis of diabetes for 4-6 years. Three of the parents did not have an adolescent participating in the focus group.

### Procedure

**Figure 2:**
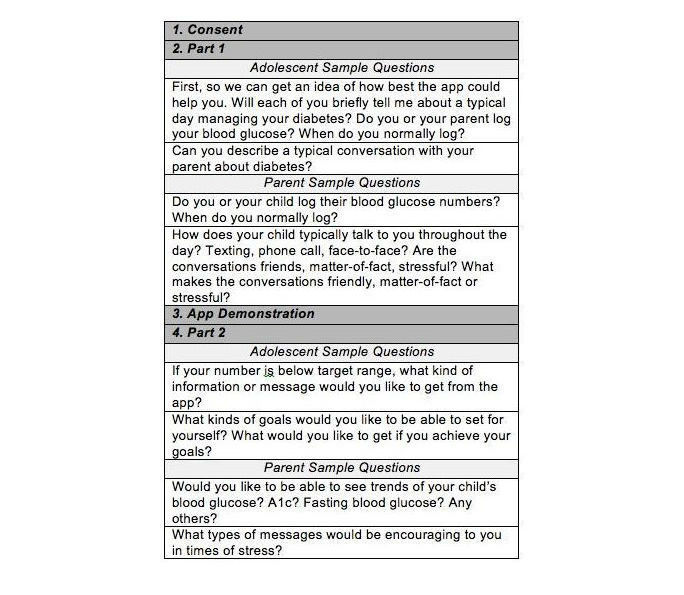
Focus group protocol content.

Figure 2 summarizes the content of the focus group protocol. Parent and adolescent focus groups were conducted separately. The adolescent focus group comprised 5 individuals, which included 4 males with a mean age of 11.6 years old (range 10-13 years), and the adolescents had a diagnosis duration of 4-6 years. The parent focus group comprised 7 individuals and included 6 females. They ranged in age from 35 to 60 years, all white, two had some college, two had a bachelor’s degree, and two had master’s degrees. The average income based on the participants’ zip code was US $80,902.20 (range: US $46,649 to US $112,774). They all resided in southeast Michigan.

Each group consisted of two sections; in the first, participants were asked about their daily diabetes management routine, how their child’s diabetes is managed (ie, amount of child responsibility), and overall communication with each other, including how often they communicate, the tone or feel of the communication (eg, positive, happy, nagging), and the channel in which they communicate (eg, phone, text, face-to-face). Gaining the perspectives of both parent and adolescent is key to designing a patient-centered app that fits the context of disease management from the point of view of those directly affected.

Additionally, we asked about current apps that they used for both diabetes management and for general entertainment. For the second portion of the focus group, the participants were given a demonstration of our app prototype. We asked them general questions about the app, what they liked about it, what they would change, and what would motivate them to use it. The parent focus group lasted for 107 min and the adolescent focus group lasted for 72 min.

### Data Analysis

All of the data collected in the focus groups were audio-recorded, transcribed verbatim, and coded using NVivo version 11 (QSR International Pty Ltd) software. Using inductive thematic analysis, part of the team iteratively developed a set of themes that captured the focus group dialog (BH, DH, AH, KM, and MK) [38]. The codes were presented to the whole group for clarification and feedback. Then, two members of the team (KM and MK), coded a random selection of the transcripts to ensure reliability. Once reliability was obtained, each transcript was coded independently by the coders. They then came together to compare and evaluate the codes. Disagreements were resolved through discussion.

## Results

### Key Themes

Four key themes were identified. These included (1) diabetes is unpredictable, (2) negative and frustrated communication, (3) motivations to use an app, and (4) feedback specific to our app. The following section describes each theme, along with descriptive quotes.

### Part 1

This part of the focus group asked participants about how they manage their diabetes and general communication around diabetes. We believe that this is key in understanding how our app could help them in their daily lives.

#### Diabetes Is Unpredictable

We first asked participants about their personal experience with diabetes and how communication about diabetes occurs between the parent and adolescent. From this line of questioning, two subthemes emerged: (1) Daily care is not an “exact science,” and (2) the transition of care increases the unpredictability. When discussing daily diabetes care, the parent participants discussed at length the importance of having good control of their child’s disease. Strategies that were successfully used for glycemic control on one day did not necessarily work another day. One parent stated:

There’s no exact science to it, it’s all a guessing game, and so it gets pretty frustrating.

Another parent stated:

You know, like, it isn’t an exact science and sometimes all those numbers that I write down—there’s too many factors to play into it.

A third parent echoed those statements and said:

We have six years of paper records...a constant deluge of information. It’s information that somebody should be able to use but it doesn’t get used because it’s not an exact science.

Because of this unpredictability, parents frequently measure their child’s blood glucose level 8 or more times per day and even have to wake up in the middle of the night to check their child. One parent reported:

I think we had two episodes of her going almost dangerously low and almost going into shock at night so that definitely sensitized us to be concerned at night. We used to get up two, three times a night and check her.

Additionally, the parents stated they often worry about their child because they have seen how fast a change in blood glucose can occur. One parent told us:

There was no warning or anything (regarding low blood glucose) and I know (it) could just go that fast from (normal)—it does happen quickly.

The transition of diabetes care from the parent to the adolescent also causes some unpredictability. The adolescents had a good understanding of the required treatment; however, they too expressed feeling overwhelmed with the amount of information to consider and the uncertainty about knowing what to do all the time. One adolescent stated:

I do a lot of it (diabetes management), but sometimes I get confused on the carbs, so I have to look it up or have someone to help me out for the day.

Another followed up:

It’s hard to do and I don’t always know what to do.

One of the parents commented on this transition saying:

They want to be independent and yet they need to be reminded. They forget, almost, that this is their issue. And again, they want to be independent but they want to be boys too.

#### Negative and Frustrated Communication

The unpredictable nature of changes in blood glucose often induces parental frustration, which leads to poor quality communication with their child. We noted that when the parent was describing their communication style, they seemingly talked at, rather than with, their child. For instance, one parent said that she says to their daughter:

You’ve got to start taking responsibility for this. It’s either going to be you or it’s going to me. And I know that you don’t want it to me be, so get on track, and start hitting this like you’re supposed to. It’s trying to get her to think about it.

Another parent echoed this by saying:

You take care of it or I do. And you know what it means when I have to take care of it. It’s a lot more nagging.

One parent said of her child attending a sleepover:

My conversation with her was, ‘I’ll get a phone call or text from you at three a.m., right? Yes, I will. And what will happen if I don’t get it? I’ll be pounding on the door.’ We’ve had to do that, so that conversation sometimes has been not so good.

All of the parents said that they knew they were frequently asking their child for blood glucose numbers and about testing.

None of the parents talked about using any strategies to communicate in a positive manner with their child surrounding diabetes management. This is not to say that they are unaware of this issue. When we asked about this, one mom said:

They are doing a great job. I mean, just the word thanks. I don’t think we say it enough.

They have good intentions to communicate positively with their child about diabetes, but the need to have their child’s blood glucose numbers overshadows any conscious effort at communicating positively.

### Part 2

In this section of the focus group, we asked them about their current use of apps to help manage diabetes and what keeps them using technologies. We then conducted a demonstration of our app prototype and gathered feedback.

#### Use of Apps to Manage Diabetes

None of the parents or the adolescents currently used an app for diabetes management or carb counting. The only technology that the parents used were programs for pump or meter downloads, like Diasend, and they generally only looked at those before attending a doctor’s appointment. One parent said:

It’s like oh crap, we have a doctor’s appointment tomorrow. Give me your pump and let me download.

Another parent said she gives their health care provider their username and password, so they could access their child’s information. Parents did, however, express that having these downloads at least once a week would be helpful.

#### Motivation

Overall, the respondents mentioned three key motivations for the use of a health-tracking app. These motivation themes included customization, interactivity, and tangible rewards.

Customization included being able to put in favorite foods, positive or affirmative messages, glucose ranges, and message types and times. Customization spurred many ideas of how and why they would use the app. They wanted to be able to enter favorite foods or restaurants to get the nutritional information. One mom said:

(I’d) put fruit roll-ups on there because it’s not in the (diabetes education) book.

Another idea included being able to customize some of the messages that the app sent, to both the child and the parent. A mother suggested being able to add in your own reminders. She said:

Would it be possible, to type in your own message? Like, say you want to write a Bible verse that’s encouraging or uplifting or something like that, or your own motto or something.

The timing of the testing and the reminders the app set seemed to be a key. One parent stated:

What if the reminders—what if you could set whatever time you wanted the reminder to be instead of having them automatic.

Another parent went on to say:

If you (allow a test within) a range...They might not be in a specific space where they can drop everything and test. It does take a minute or two to actually test.

Interactivity was another motivation suggested by the participants. This refers to not wanting to simply be told information, but to be able to interact with the app in interesting and meaningful ways. For instance, adolescents like the idea of being part of a team. In a popular app-based game that the adolescents played, they worked in teams or clans to defend villages and farms. Thus, they were especially keen on being able to join a team to earn points. One adolescent stated:

Everyone who uses the app has a team and the team with the most points wins.

They also liked the idea of getting individual points in order to get virtual character badges, new “powers” or tools, clothing, and or accessories. One adolescent suggested:

If you get a bunch of points, you could have a character with like a hat and sunglasses or something.

Along with those intrinsic motivations, tangible rewards like earning points redeemable for purchases made at the Apple iStore, Google Marketplace, or Amazon were very popular. For example, a parent said:

The Amazon gift card thing—for mine because they are teenagers and are like—Amazon is the best thing.

One of our adolescents echoed this sentiment stating, “Maybe get points on Amazon.” The adolescents were excited about the idea of getting points through the app. One suggested that the more a person opened the app and used the app to enter blood glucose values, the more points would be earned. One adolescent stated that they would also like to be able to redeem the points with their parents.

You could use the points for a day of no nagging (from the parents).

#### App Feedback

One of the key features of this app was to show parents that their children have tested their blood glucose, but not to show the number immediately. This is designed to increase trust, self-efficacy, and autonomy to help the transition process. We asked both the parents and the adolescents specifically about this feature. All of them said that the parent needs to know the test number immediately. One stated:

His numbers are all over the place. You know, I wouldn’t feel comfortable with that (not seeing the numbers right away).

The parents were also aware that they have difficulty in relinquishing control over their child’s diabetes care management. For example, one mom said:

I mean, it’s nice to have a break as a parent but I can’t have a break right now.

The adolescents mostly agreed that the parent should see the number, but more as a way to move the conversation from face-to-face to the app. One adolescent stated:

They should be able to see the low and the high, but they don’t need to see the regular number. Because they’re just like—check their phone, like oh, she’s alright.

Both groups gave positive reviews of the overall app as well as the different features within the app. One mom thought that having the app mediate the communication would be very helpful. Rather than the parent constantly telling the child what to do, the app would remind them to stay on top of their management, allowing more time for the parent to communicate with their child about things other than diabetes. The parents also suggested the use of emojis; one said:

I think you could also have emojis that are sad faces or tears or like a heart.

One of the adolescents repeated this idea stating:

I think like maybe if they (parents) could text you an emoji thumbs up that would be good.

Another said:

(The kids) lie because they don’t want you to be disappointed, they don’t want you to be mad, they don’t want you yelling at them. So I think this (the app)—doing it this way would take that whole scenario out of it.

## Discussion

### Principal Findings

mHealth used for diabetes management is growing as a method to improve adherence and health outcomes. Using a patient-centered approach, this study sought to understand parent and adolescent perspectives of on-going development of a T1D app. To fully understand how an app could be engaging to both the parent and the child, it was important to understand the environment in which an app would be used. To achieve this goal, we asked parents and adolescents to tell us about how they currently manage the child’s diabetes and how they communicate about the disease. We then showed them a demonstration of our prototype app to obtain their perspectives on the app and additional components they thought the app should have.

These focus groups acknowledge the complexity of disease management and the frustrations in communication for families with an adolescent with T1D. Parents feel the need to remain in control because the day-to-day management of diabetes is very unpredictable. Consistent with other research, the unpredictability fuels some of the frustration that the parent and the adolescent feel and ultimately leads to inadequate communication strategies [23]. Our proposed app is designed to help improve the communication between the parent and their child. The app helps monitor the adolescent’s blood glucose and provides positive message prompts to the parent for better communication.

In order for an app to be successful and provide benefit to users, people must actively use it. We asked about different strategies that would keep the users engaged with the app. Customization, interactivity, and tangible rewards were discussed as motivating factors of using the app [37]. This is similar to other studies that have concluded that customization is important to appeal to the needs of various users and that it is imperative to discuss what options should be customizable [39-41]. Having the app be interactive was also stated as a motivation for engaging and using the app. Currently, there is limited interactivity in the downloads of their meter or their pump, which could be one cause of their low usage. Past research has demonstrated that having interactive functions have improved usability and resulted in higher engagement [42-45]. Through these focus groups, we have decided to integrate more interactivity in the app through some of the customization features, as well as through how the parents and child communicate through the app.

Participants also agreed that providing tangible rewards or financial incentives would motivate users to remain engaged with the app. Some research has shown that financial incentives are key motivators in weight loss and smoking cessation [46]. Ethical issues remain related to incentivizing individuals in this manner and what this practice might mean particularly for disadvantaged populations [47]. Additionally, there are issues of long-term sustainability both in terms of the health impacts and how the program would be able to continue to provide those incentives [48]. Because of these issues, we have decided to use some gamification techniques to reward use of the app. The utilization of gamification seeks to encourage users to complete specific tasks within a nongame environment [49-51]. There are several ways to do this, which include giving points every time a user opens the app, and having participants earn badges or points to “buy” items for avatars. Past research has suggested that having an environment that is enjoyable to use can increase health knowledge, efficacy, and adherence to their daily care can improve health outcomes [52-56].

A striking feature from the parent focus group was how parents reported communicating with their child about diabetes. In the current communication cycle, parents often report becoming frustrated and upset with their child, which does not help them learn how to handle the issue in a positive manner [57]. This may lead to the adolescent lying to their parents in hopes of preventing them from becoming frustrated or upset. This leads to further deterioration in the quality of the parent-child communication. In short, the parents talk at their adolescent, commanding them to pay attention to their care rather than working with them to successfully manage diabetes. Therefore, we believe adding problem-solving resources and features to the app would be beneficial. Problem solving, defined by Yeates and colleagues [58], is conceptualized as defining a problem, generating alternative strategies, evaluating outcomes, and selecting a strategy. Some literature has demonstrated positive associations between problem-solving behaviors and improved HbA1c; however more research is needed in this area [59].

An inherent feature of technology is its distance between users. Past research has suggested that “online interaction reduced embarrassment that might be experienced in face-to-face interactions and thus encourages self-disclosure” [60]. There are many studies demonstrating that computer mediated communication enhances self-disclosure [61-62]. This is especially important for adolescents who, developmentally, are often hyper self-aware and self-conscious [62]. As the proposed app will notify parents that their child has measured their blood glucose level, and in some cases report what the blood glucose level is, parents will be able to respond with affirmative preprogrammed messages through the app. We believe that the outcome of app use will change the dynamic of the communication. The adolescent will be more likely to provide true numbers and the parents will have preprogrammed messages with which they can respond, thereby making communication less frustrating and a more honest and constructive exchange.

One of the desirable app features that the adolescents mentioned was to join a team. A growing number of researchers are starting to focus on social interaction in mobile apps. Although fun is the primary reason many use apps, social interaction was reported as the second reason to play games [63]. Using technology in a group leads to greater enjoyment, lower dropout rate, and longer engagement compared with individual play [64]. It has been shown in health and sports studies that most people enjoy playing in a group more than by themselves [65]. Additionally, past research has demonstrated improvement in glycemic control for children with T1D can result from the responsibility of caring for others [66]. Researchers hypothesized that the structured care provided cues that translated to the performance of diabetes self-management behaviors. This suggests that by caring for another, possibly through a team setting, individuals learn to care better for themselves.

Implications for other groups developing an app for children with a chronic disease and their parent include understanding the current communication context that disease management takes place in. As a result of this insight, we were able to add a problem-solving framework into our app. We were also able to understand the importance of having the messages and reminders come from the app and not the parent. Team participation within the app appears to be a way to keep the adolescents engaged and responsible for their own care. Finally, we learned that some type of reward needs to be added. To achieve this goal, we have added the ability for the adolescent to purchase different accessories for their in-app avatar. Without understanding the communication that currently takes place between the parent and the child, we would have not been able to make these adjustments.

Whereas there are some limitations to this work, the goal was to gain a patient-centered perspective on the app design to date. These focus groups are not representative of all mHealth apps, as ours is specifically for adolescents with T1D and includes their parent. Additionally, our sample size was small. However, we believe that this work provides information that may help others in their app development process. Future studies of this type should include larger samples, including a wider age range of youth.

### Conclusions

This app has the potential to provide parents the security they need to start transitioning diabetes management to their children and allowing them to develop the confidence needed for ongoing optimal control of their diabetes. This study used an original app idea and demonstrated a prototype of the app to help guide both the parents’ and the adolescents’ perspectives to design this patient-centered diabetes app. Overall, the app review was positive and the participants thought it would be useful to aid in the transition to adolescent self-management. Understanding the needs of patients and caregivers will help better inform the development and design of the app. This study also provides others with strategies that can be incorporated into the development of other health apps, including use of a patient-centered design, incorporating interactivity, use of customization, and building teams within the app.

## Abbreviations

ADA: American Diabetes Association
HbA1c: glycosylated hemoglobin
JDRF: Juvenile Diabetes Research Foundation
T1D: type 1 diabetes
